# Admission C-Reactive Protein and Mortality After STEMI: A Retrospective Cohort Study Identifying Subgroup-Specific Risk Thresholds

**DOI:** 10.3390/jcm15082864

**Published:** 2026-04-09

**Authors:** Kristen Kopp, Magdalena Leitner, Nikolaus Clodi, Michael Lichtenauer, Matthias Hammerer, Uta C. Hoppe, Elke Boxhammer, Mathias C. Brandt

**Affiliations:** Department of Internal Medicine II, Division of Cardiology, Paracelsus Medical University of Salzburg, 5020 Salzburg, Austria

**Keywords:** ST-segment elevation myocardial infarction, C-reactive protein, inflammation, mortality, risk stratification

## Abstract

**Background**: Inflammation is central to myocardial injury and repair after ST-segment elevation myocardial infarction (STEMI). C-reactive protein (CRP) is an established biomarker of systemic inflammation, but its prognostic thresholds across patient subgroups are not well defined. **Methods:** In this retrospective cohort study, admission CRP was analyzed in 958 consecutive STEMI patients admitted to University Hospital Salzburg 2018–2020 and categorized into four groups (Serum CRP < 5.0, 5.0–9.9, 10.0–15, and >15.0 mg/dL). Mortality was assessed during short- (30, 90, and 180 days) and long-term (1, 3, and 5 years) follow-up. Kaplan–Meier analyses compared survival, Cox regression tested associations, and receiver operating characteristic (ROC) curves determined discriminatory value and optimal cut-offs. **Results:** Elevated admission CRP was associated with larger infarct size, impaired left ventricular function, and increased mortality. Kaplan–Meier curves showed progressively poorer survival with higher CRP, with worst outcomes at >15 mg/dL. At 30, 90, and 180 days, CRP demonstrated moderate discrimination (AUC 0.628, 0.653, and 0.654; all *p* < 0.001), with predictive cut-offs 11–15 mg/dL in the overall cohort. Subgroup analyses revealed markedly lower thresholds in vulnerable populations. Diabetic patients showed cut-offs 5–6 mg/dL with the highest AUC values (up to 0.714). Younger patients and smokers exhibited thresholds near 9–10 mg/dL, while subacute STEMI presentations demonstrated lower cut-offs compared with acute infarction. These findings indicate that the prognostic value of CRP is context-dependent rather than uniform. **Conclusions:** Admission CRP predicts short-term mortality after STEMI, with subgroup-specific cut-offs emerging below conventional thresholds, highlighting profiles where modest inflammatory activation carries disproportionate risk.

## 1. Introduction

When a patient presents with crushing chest pain, the ECG leaves little doubt: ST-segment elevations signal an ongoing myocardial infarction. Reperfusion therapy is an urgent priority, and advances in catheter-based interventions have transformed survival rates over the past two decades. Yet even after timely treatment, outcomes vary widely. Some patients recover fully, others develop progressive heart failure, and some die within days or months. This variability prompts an important question: what factors, beyond coronary anatomy, prompt management and procedural success, shape prognosis after STEMI?

Increasing evidence points toward the role of systemic inflammation in this process. The rupture of an atherosclerotic plaque and the ensuing myocardial injury trigger a cascade of inflammatory mediators and immune cell activation [1]. Necrotic cardiomyocytes release danger-associated molecular patterns (DAMPs), activating innate immune pathways and inducing the release of pro-inflammatory cytokines such as interleukin-6 (IL-6) [2]. While this immune activation is necessary for tissue repair, excessive or prolonged inflammation can exacerbate myocardial damage, promote maladaptive ventricular remodeling and destabilize non-culprit plaques [3].

C-reactive protein (CRP) is a well-characterized acute-phase reactant and one of the most widely used clinical biomarkers of systemic inflammation. Synthesized predominantly by the liver under IL-6 stimulation, CRP rises rapidly after an acute coronary event, typically peaking within 48–72 h [4]. Elevated CRP levels have been linked to adverse cardiovascular outcomes in both chronic and acute coronary syndromes. In stable populations, high-sensitivity CRP predicts incident myocardial infarction, stroke and cardiovascular death, independent of conventional risk factors [5,6]. In acute coronary syndromes, several cohort studies have reported that higher CRP concentrations on admission or early after infarction are associated with larger infarct size, reduced left ventricular ejection fraction and increased mortality [7,8].

In STEMI specifically, observational studies suggest that elevated baseline CRP is associated with greater in-hospital complication rates and short- [9] and long-term mortality [10]. However, findings are not entirely consistent. Some analyses have demonstrated that CRP remains an independent predictor of mortality after adjusting for established risk factors [11,12], whereas others have found its prognostic significance attenuated when accounting for age, Killip class and renal function [13]. Variability in CRP measurement timing, assay sensitivity and patient selection may partially explain these discrepancies. Furthermore, most prior studies have concentrated on short-term outcomes, leaving the relationship between admission CRP and long-term mortality insufficiently defined.

Clarifying this association is clinically relevant for several reasons. CRP is inexpensive, universally available, and rapidly measurable, making it a practical adjunct to existing risk scores. Understanding the prognostic role of systemic inflammation could also inform the design of targeted anti-inflammatory strategies, as highlighted by the CANTOS trial, which demonstrated event reduction with IL-1β inhibition in patients with prior myocardial infarction and elevated CRP [14].

The present study investigates the relationship between admission CRP levels and all-cause mortality regarding short- and long-term prognosis in a large, well-defined cohort of STEMI patients. We examine survival across predefined CRP categories and analyze CRP as a continuous variable to capture potential dose–response effects. Kaplan–Meier methods are used for unadjusted comparisons, and multivariable Cox regression models assess the independent predictive value of CRP after adjustment for demographic, clinical, and procedural variables. By integrating short- and long-term follow-up, this study aims to provide a comprehensive assessment of the prognostic relevance of CRP in STEMI, contributing to a more refined understanding of how systemic inflammation influences post-infarction outcomes.

## 2. Materials and Methods

### 2.1. Study Population

All patients (*n* = 964) presenting with ST-segment elevation myocardial infarction (STEMI) at the University Hospital Salzburg, a large tertiary care center in Austria, between 1 January 2018 and 31 December 2020 were screened for this retrospective observational study. Inclusion criteria were age ≥ 18 years, a confirmed diagnosis of STEMI according to criteria outlined in The Fourth Universal Definition of Myocardial Infarction [15], and availability of CRP values obtained during the index hospitalization. Six patients were excluded due to concomitant severe infection at admission, given the potential confounding effect on CRP levels. The final study cohort consisted of 958 patients.

### 2.2. Ethics Statement

The study protocol was approved by the State of Salzburg Ethics Commission (EK-Nr. 1038/2021). Patient consent was waived by the Ethics Commission due to the retrospective design of the study. All data handling was in compliance with the principles outlined in the Declaration of Helsinki and Good Clinical Practice (ICH-GCP) guidelines.

### 2.3. Data Collection

Clinical and laboratory data were obtained from the hospital’s electronic medical record system (ORBIS, Agfa Healthcare, Version 08043301.04110DACHL) and the medical archiving system (Krankengeschichtsarchiv System 2008, Uniklinikum Salzburg, Softworx by Andreas Schwab^TM^, Salzburg, Austria). Information from admission and discharge reports, laboratory results, and procedural documentation from the index hospitalization was extracted. All data were pseudo-anonymized before analysis and stored in a secure database.

### 2.4. Laboratory Measurements

All laboratory analyses were performed at the University Institute for Medical-Chemical Laboratory Diagnostics, University Hospital Salzburg. CRP concentrations were measured in serum samples using an immunoturbidimetric assay on the c702 module of the Roche Cobas^®^ 8000 analyzer (Roche Diagnostics, Mannheim, Germany), following the manufacturer’s instructions.

### 2.5. STEMI Diagnosis

STEMI was diagnosed based on clinical symptoms consistent with myocardial ischemia, characteristic electrocardiographic changes (ST-segment elevation in ≥2 contiguous leads of ≥2 mm in men or ≥1.5 mm in women in leads V2–V3 and/or ≥1 mm in other contiguous chest or limb leads), and elevated cardiac biomarkers (e.g., high-sensitivity troponin T), in accordance with criteria outlined in The Fourth Universal Definition of Myocardial Infarction [15], the joint guideline in place during the study period. In our study, subanalysis of acute and subacute STEMI was performed. Acute STEMI was defined as <12 h from STEMI diagnosis to reperfusion, while subacute STEMI was defined as 12 h- 48 h from STEMI diagnosis to reperfusion, in accordance with the European Society of Cardiology (ESC) guidelines in place at the time of the study [16]. Patients with recent STEMI defined as >48 h from STEMI diagnosis to reperfusion were excluded from the study.

### 2.6. CRP Categorization

Standard CRP assays were routinely measured in mg/dL in patients presenting with STEMI at our clinic. Measurement of high-sensitivity CRP (hs-CRP) was not routinely performed in our patient population due to cost and availability and due to conservative European Society of Cardiology Class IIb, level B recommendations in place at the time of the study observation period [17]. Please refer to the Limitations section, also, as only single CRP measurements were done at the time of STEMI admission.

For primary analyses, patients were categorized into four groups according to admission CRP values standardly measured in mg/dL:

- <5.0 mg/dL;
- 5.0–9.9 mg/dL;
- 10.0–15.0 mg/dL;
- >15.0 mg/dL.

These categories were chosen a priori based on the prior literature linking low-grade and high-grade inflammation to cardiovascular outcomes [18,19,20]. CRP was also analyzed as a continuous variable (z-transformed) in sensitivity analyses to assess linearity.

### 2.7. Outcomes

The primary outcome of this study was overall all-cause mortality following the index STEMI event. Survival time was calculated from the date of hospital admission for STEMI, with a maximum follow-up duration of 60 months. Mortality data were obtained from hospital medical records, the Austrian national death registry, follow-up communication with primary care physicians, and, when necessary, direct contact with family members. All deaths, irrespective of cause, were included in the analysis.

### 2.8. Statistical Analysis

Statistical analyses were performed using SPSS Version 25 (IBM SPSS Statistics, Armonk, NY, USA). The Kolmogorov–Smirnov–Lilliefors test was applied to assess the normality of continuous variables. Normally distributed variables were expressed as mean ± standard deviation (SD) and compared using one-way analysis of variance (ANOVA); non-normally distributed variables were reported as median (interquartile range, IQR) and compared using the Kruskal–Wallis test. Categorical variables were presented as frequencies and percentages, with between-group comparisons made using the chi-square test.

Survival probabilities were estimated using Kaplan–Meier curves, with differences between CRP categories evaluated by the log-rank test. Cox proportional hazards regression models were used to evaluate the association between admission CRP categories and short-term mortality, using CRP < 5.0 mg/dL as the reference category. To explore potential effect modification and assess the robustness of the association, subgroup analyses were performed across clinically relevant patient groups, including STEMI presentation (acute vs. subacute), BMI, smoking status, diabetes, age, and sex.

Proportional hazards assumptions were verified using Schoenfeld residuals. Hazard ratios (HR) are reported with corresponding 95% confidence intervals (CI).

To further assess the discriminatory ability of admission CRP for predicting short-term outcomes, area under the receiver operating characteristic (AUROC) curve analyses were performed at 30, 90, and 180 days. Areas under the curve (AUC) were calculated with 95% CI, and the optimal cut-off values were determined using the Youden index (YI). For each subgroup analysis (sex, age, diabetes, smoking status, BMI, and STEMI presentation), AUC values, cut-offs, sensitivity, specificity, and YI were derived. These analyses were restricted to short-term mortality because CRP represents an acute-phase inflammatory marker, with its strongest prognostic impact expected in the early post-infarction period.

A two-sided *p*-value ≤ 0.05 was considered statistically significant.

## 3. Results

### 3.1. Baseline Characteristics of Study Cohort

The study population was comprised of 958 patients with STEMI, stratified into four groups according to admission CRP levels (<5.0 mg/dL, 5.0–9.9 mg/dL, 10.0–15.0 mg/dL, and >15.0 mg/dL). Baseline characteristics are summarized in Table 1.

The mean age of the cohort was 63.4 ± 12.0 years and increased modestly across CRP groups. Diabetes was more frequent in patients with high CRP (24.1% in > 15.0 mg/dL vs. 14.0% in 5.0–9.9 mg/dL), whereas dyslipidemia was less common (54.7% vs. 72.4%). Markers of more severe presentation clustered in the highest CRP group, with cardiogenic shock in 48.2% and cardiopulmonary resuscitation in 40.1%, compared with 6.3% and 6.1% in the lowest CRP group. Our STEMI cohort had a high percentage (17%) of patients with diagnosis of cardiogenic shock associated with tissue damage, global severity, hypoperfusion and inflammation [21] and/or respiratory failure requiring intubation. To note, cardiogenic shock was classified using SHOCK criteria at our clinic, comprising systolic blood pressure SBP < 90 mmHg for ≥ 30 min or use of pharmacological and/or mechanical support (IABP) to maintain an SBP ≥ 90 mmHg [22]. Among our cardiogenic shock patients < 1% were managed with extracorporeal membrane oxygenation (ECMO) and the percentage of IABP management, although more common, was not captured in our database. Impella is not in use at our center. Infarct size was larger in patients with elevated CRP, reflected by higher peak troponin T (4350 ng/L vs. 3031 ng/L) and creatine kinase (2278 U/L vs. 1195 U/L). Left ventricular ejection fraction declined accordingly, from 45.6% in patients with CRP < 5.0 mg/dL to 37.8% in those with CRP > 15.0 mg/dL.

### 3.2. Kaplan–Meier Survival Analysis by CRP Category

**Figure 1:**
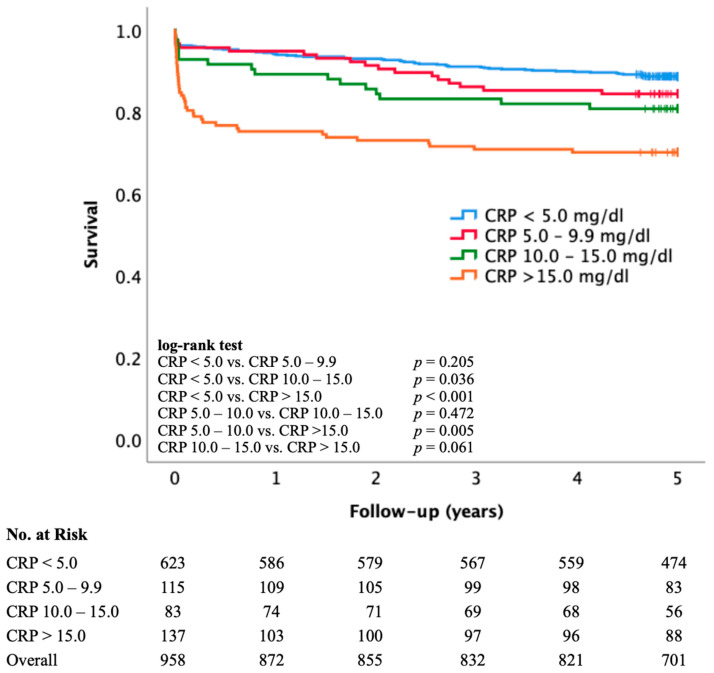
Kaplan–Meier survival curves according to admission CRP categories. Key: Long-term overall survival in STEMI patients stratified by admission CRP levels: <5.0 mg/dL (blue), 5.0–9.9 mg/dL (red), 10.0–15.0 mg/dL (green), and ≥15.0 mg/dL (orange). Survival decreased progressively with higher CRP levels, with the poorest outcomes observed in the ≥15.0 mg/dL group. Pairwise *p*-values from log-rank tests are shown in the inset table. CRP = C-reactive protein; mg/dL milligram per deciliter.

Kaplan–Meier analysis demonstrated a stepwise decrease in long-term survival with increasing admission CRP concentrations (Figure 1). Patients with CRP < 5.0 mg/dL showed the highest survival probability over the 5-year follow-up, whereas those with CRP > 15.0 mg/dL had the poorest outcomes, with an early and sustained separation of survival curves. Pairwise log-rank testing revealed that survival in the CRP > 15.0 mg/dL group was significantly lower compared with all other groups (all *p* < 0.006), except for the CRP 10.0–15.0 mg/dL group (*p* = 0.061). Patients with CRP 10.0–15.0 mg/dL also exhibited significantly reduced survival compared to the CRP < 5.0 mg/dL group (*p* = 0.036), whereas no significant difference was observed between CRP < 5.0 mg/dL and CRP 5.0–9.9 mg/dL (*p* = 0.205) or between CRP 5.0–9.9 mg/dL and CRP 10.0–15.0 mg/dL (*p* = 0.472). These findings indicate that the prognostic impact of elevated CRP is most pronounced at concentrations exceeding 15.0 mg/dL, with intermediate values showing a graded but less distinct risk profile.

### 3.3. Univariate Cox Regression According to CRP Categories—Short-Term Mortality

In the overall STEMI cohort, admission CRP > 15.0 mg/dL was strongly associated with increased short-term mortality at all examined intervals. Compared with patients with CRP < 5.0 mg/dL, the hazard ratio reached 4.29 at 30 days, increased to 5.32 at 90 days, and remained elevated at 5.17 at 180 days, each within the bright red range of the color code and statistically significant.

This excess risk for CRP > 15.0 mg/dL was observed consistently across most subgroups, including both sexes, smokers and non-smokers, higher and lower BMI categories, and both acute and subacute STEMI presentations. The effect was most pronounced in patients with diabetes, where hazard ratios peaked at 11.12 at 90 days (dark red) and remained substantially elevated at 30 and 180 days.

In contrast, the CRP 10.0–15.0 mg/dL category demonstrated a much more restricted prognostic impact. Significant associations were limited to patients younger than 70 years and to those with diabetes. In younger patients, hazard ratios remained within the red range across all short-term intervals, exceeding fourfold increases in mortality risk. In diabetic patients, hazard ratios ranged from 5.80 at 30 days to 7.75 at 90 days, remaining above 5 at 180 days, all in the bright red to red range. No other subgroup reached statistical significance for this intermediate CRP category, and CRP 5.0–9.9 mg/dL was not significantly associated with short-term mortality in any analysis.

**Figure 2:**
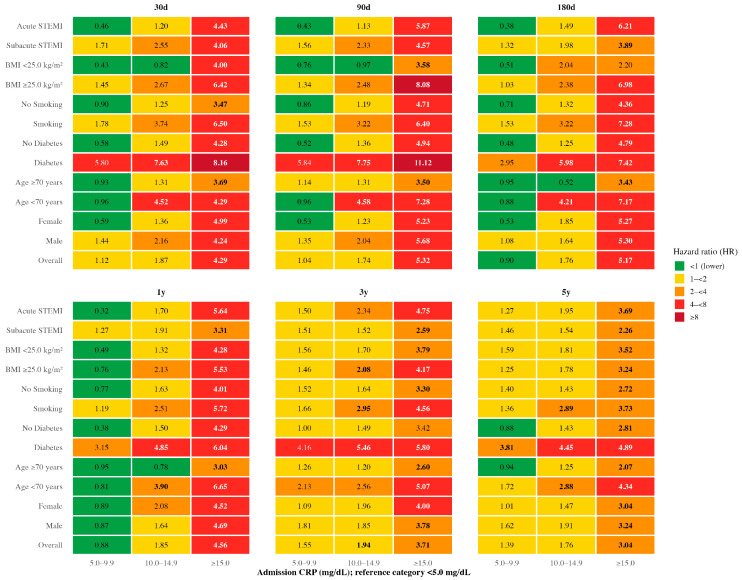
Cox regression analysis of admission CRP and mortality after STEMI across clinical subgroups. Heatmaps displaying hazard ratios (HR) derived from Cox proportional hazards regression models across clinically relevant subgroups. Admission CRP was analyzed in predefined categories (5.0–9.9, 10.0–14.9, and ≥15.0 mg/dL), with CRP < 5.0 mg/dL as the reference category. Panels represent different follow-up intervals (30, 90, and 180 days; 1, 3, and 5 years). HRs are shown for each subgroup, including STEMI presentation (acute vs. subacute), body mass index, smoking status, diabetes, age, and sex. Estimates represent Cox regression models calculated within each subgroup using predefined clinical covariates. Color coding reflects the magnitude of the hazard ratio (<1, 1–<2, 2–<4, 4–<8, and ≥8) and is intended for visual comparison across subgroups and time points. Confidence intervals are not displayed to preserve figure readability given the large number of subgroup–timepoint combinations. Subgroup definitions and sample sizes are provided in Table 1. Bold font denotes statistical significance (*p* < 0.05) compared with the reference. CRP = C-reactive protein; mg/dL = milligram per deciliter; BMI = body mass index; STEMI = ST-segment elevation myocardial infarction.

Overall, these findings highlight that markedly elevated CRP levels at admission identify a broad high-risk phenotype for early mortality following STEMI, whereas intermediate elevations confer additional risk only in younger patients and those with diabetes. The color-coded heatmap in Figure 2 visually emphasizes this risk gradient, with clusters of bright and dark red confined to these highest-risk profiles.

### 3.4. Univariate Cox Regression According to CRP Categories—Long-Term Mortality

In the overall STEMI cohort, admission CRP > 15 mg/dL was strongly associated with increased long-term mortality at 1, 3, and 5 years compared with patients with CRP < 5.0 mg/dL. The hazard ratio was highest at 1 year (HR 4.56, bright red range), declined to 3.71 at 3 years, and remained at 3.04 at 5 years, both in the yellow range and statistically significant.

This association for CRP > 15.0 mg/dL was consistent across all subgroups. The largest effect sizes were seen in younger patients, where HRs reached 6.65 at 1 year (dark red) and remained elevated at 4.34 at 5 years, and in diabetic patients, where values for CRP > 15.0 mg/dL often exceeded 4 across all follow-up intervals.

The intermediate CRP category (10.0–15.0 mg/dL) also showed significant associations in the overall cohort but with lower HRs than in the highest category. These effects were largely driven by specific subgroups; in younger patients, diabetics, smokers and those with BMI ≥ 25 kg/m^2^, this category consistently predicted higher mortality, with HRs ranging from the yellow to bright red range depending on follow-up time. In females, older patients, non-smokers, and those with lower BMI, significance was not observed for the intermediate category. The 5.0–9.9 mg/dL range showed only one statistically significant association in diabetic patients at a follow-up of 5 years.

Overall, the long-term analyses confirm that markedly elevated CRP levels at STEMI presentation are a robust predictor of mortality across a wide range of patient profiles, with the greatest relative risks occurring within the first year after the index event. Intermediate CRP elevations appear to have prognostic value primarily in subgroups with additional high-risk characteristics, such as younger age, male sex, diabetes, smoking, or overweight. The color-coded heatmap in Figure 2 (lower half) illustrates this risk gradient, with the brightest and darkest shades concentrated in the highest CRP category, particularly 1 year after STEMI.

### 3.5. AUROC Analysis for Short-Term Mortality

**Figure 3:**
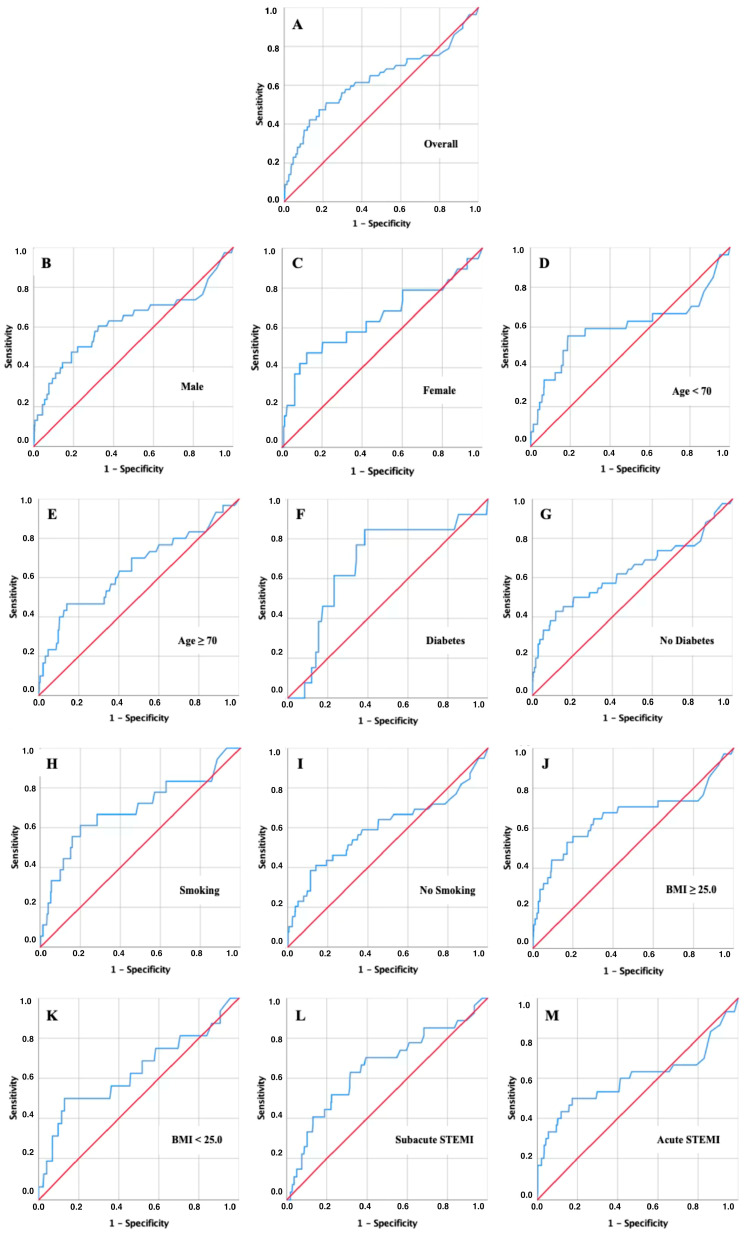
ROC curve analysis for 30-day mortality. Key: Receiver operating characteristic (ROC) curves of admission CRP for predicting 30-day mortality in the overall cohort (**A**) and predefined subgroups: Males (**B**), Females (**C**), Younger Patients < 70 years (**D**), Older Patients ≥ 70 years (**E**), Diabetes Patients (**F**), Non-diabetic Patients (**G**), Smokers (**H**), Non-smokers (**I**), Overweight/Obese Patients (**J**), Non-overweight/non-obese Patients (**K**), Subacute STEMI (**L**), Acute STEMI (**M**).

**Figure 4:**
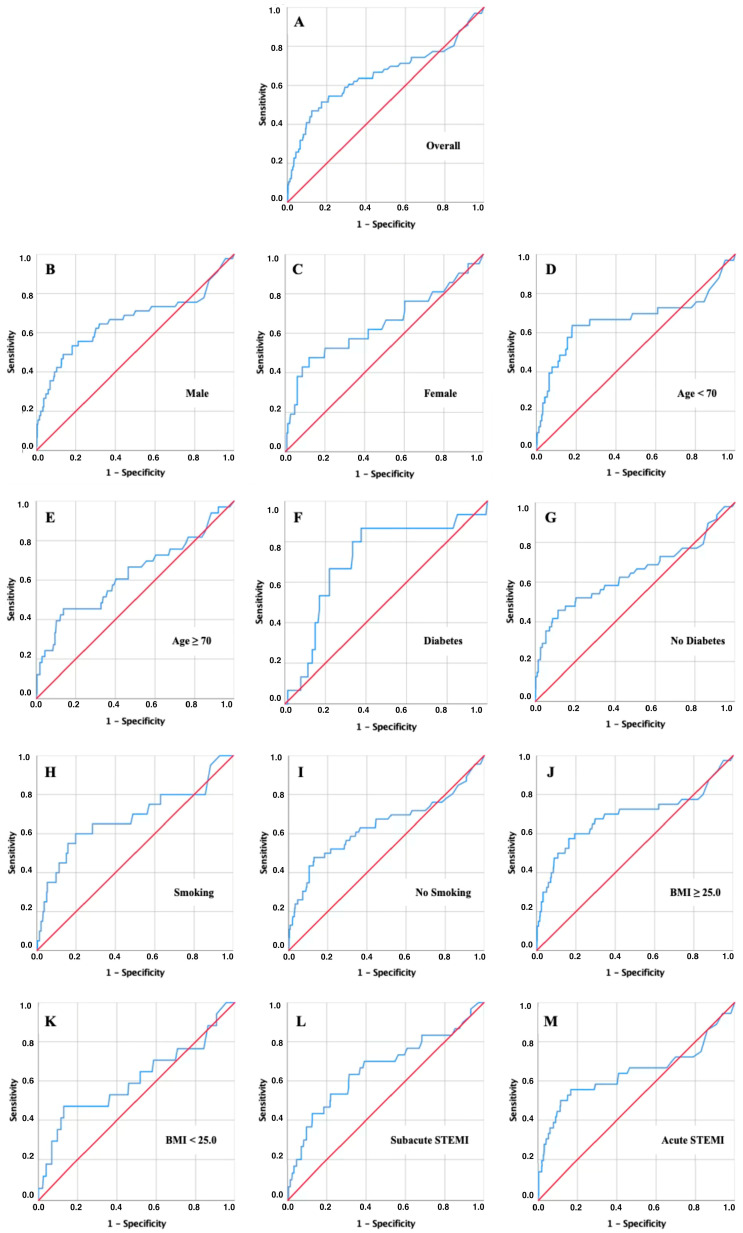
ROC curve analysis for 90-day mortality. Key: Receiver operating characteristic (ROC) curves of admission CRP for predicting 90-day mortality in the overall cohort (**A**) and predefined subgroups: Males (**B**), Females (**C**), Younger Patients < 70 years (**D**), Older Patients ≥ 70 years (**E**), Diabetes Patients (**F**), Non-diabetic Patients (**G**), Smokers (**H**), Non-smokers (**I**), Overweight/Obese Patients (**J**), Non-overweight/non-obese Patients (**K**), Subacute STEMI (**L**), Acute STEMI (**M**).

**Figure 5:**
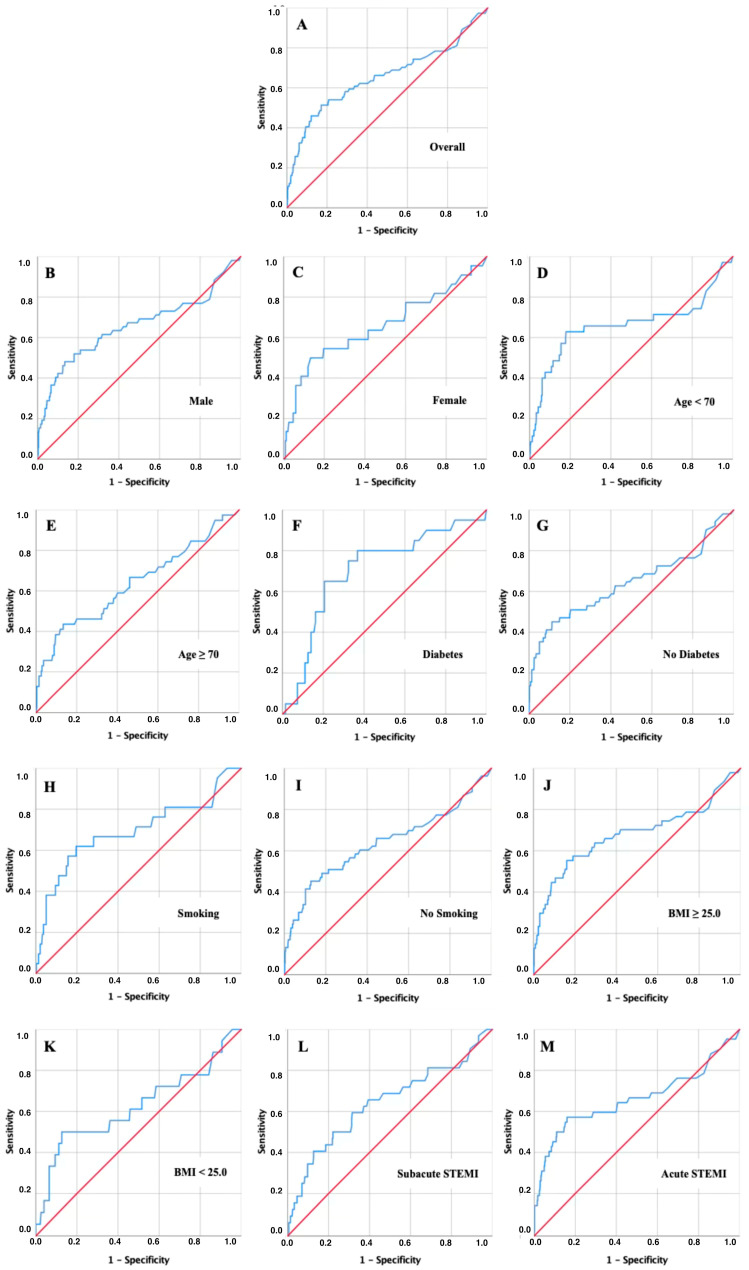
ROC curve analysis for 180-day mortality. Key: Receiver operating characteristic (ROC) curves of admission CRP for predicting 180-day mortality in the overall cohort (**A**) and predefined subgroups: Males (**B**), Females (**C**), Younger Patients < 70 years (**D**), Older Patients ≥ 70 years (**E**), Diabetes Patients (**F**), Non-diabetic Patients (**G**), Smokers (**H**), Non-smokers (**I**), Overweight/Obese Patients (**J**), Non-overweight/non-obese Patients (**K**), Subacute STEMI (**L**), Acute STEMI (**M**).

The discriminatory value of admission CRP for short-term mortality was assessed using ROC curve analyses at 30, 90, and 180 days (Figure 3, Figure 4 and Figure 5 and Table 2, Table 3 and Table 4).

In the overall cohort, CRP showed moderate discriminatory power with AUC values of 0.628 at 30 days, 0.653 at 90 days, and 0.654 at 180 days (all *p* < 0.001). The corresponding risk cut-offs were consistently in the range of 11–15 mg/dL, with relatively balanced sensitivity and specificity (Table 2, Table 3 and Table 4).

Subgroup analyses revealed, however, that several clinically relevant patient groups reached substantially higher discriminatory accuracy at lower CRP thresholds. In patients with diabetes, CRP predicted mortality with the highest AUC values across all time points (0.678 at 30 days, 0.714 at 90 days, and 0.705 at 180 days), and the optimal cut-offs clustered around only 5–6 mg/dL. A similar pattern was observed in younger patients (<70 years), who reached an AUC of 0.677 at 90 days with a cut-off of approximately 9.5 mg/dL, well below the threshold identified in the overall population. Smokers also showed increased sensitivity to modest CRP elevations, with cut-offs near 9–10 mg/dL and AUC values in the range of 0.64–0.69. Finally, patients with subacute STEMI consistently demonstrated lower cut-offs (around 9–10 mg/dL) compared to those presenting with acute infarction.

## 4. Discussion

### 4.1. What Is New: Context-Dependent Prognostic Meaning of Admission CRP

Our data reaffirm that admission CRP carries prognostic information after STEMI, but they also refine how that information should be interpreted. Beyond showing moderate discrimination for short-term mortality in the overall cohort, we observed that the prognostic threshold of CRP is not uniform. In younger patients, diabetics, smokers, and those with subacute infarction, risk emerges at substantially lower CRP values than in the general population. This pattern suggests differential vulnerability to acute inflammatory activation; modest CRP elevations—well below the conventional “high-risk” range—already identify near-term mortality risk in these subgroups, whereas the overall cohort requires higher levels to achieve comparable discrimination. This finding integrates biologically with contemporary understanding of the post-infarction inflammatory cascade. Myocardial necrosis triggers innate immune activation, leukocyte recruitment, and cytokine signaling that are indispensable for debris clearance yet can drive adverse remodeling when excessive or prolonged [23]. Frangogiannis’ synthesis of this response underscores how the same pathways that orchestrate repair also mediate injury and heart-failure progression, with toll-like receptor signaling, complement activation, and IL-1/IL-6 signaling as key nodes [2]. Westman et al. [3] similarly argue that inter-individual variation in the intensity and duration of this inflammatory burst contributes to infarct expansion and left-ventricular remodeling, providing a mechanistic bridge between an acute-phase marker like CRP and clinical outcomes.

Not only C-reactive protein (CRP) but also interleukin-6 (IL-6), interleukin-1β (IL-1β), tumor necrosis factor-α (TNF-α), white blood cell count (WBCc), and novel indices such as neutrophil-to-lymphocyte ratio (NLR) and systemic immune-inflammation index (SII) are laboratory markers of inflammation that rise in acute myocardial infarction. These markers are associated with larger infarct size, impaired left ventricular function, increased risk of arrhythmias, endothelial dysfunction, and worse prognosis, including higher short-term and long-term mortality and heart failure risk [24]. IL-6 and CRP are particularly strong predictors of adverse outcomes and heart failure post-AMI in younger patients < age 60 years, findings aligning with our observations [25]. Interleukin-6 (IL-6) is a central, pleiotropic cytokine that serves as a primary upstream mediator of the inflammatory response during acute myocardial infarction (AMI) [26,27]. It is rapidly elevated following the acute ischemic event, correlating with the severity of tissue injury, and is a major determinant of downstream acute-phase reactants like C-reactive protein (CRP) [28,29].

In the setting of AMI, IL-6 is produced by various cells, including macrophages, endothelial cells, and specifically activated cardiac fibroblasts [26,28]. While a transient rise may support early tissue repair, persistent or excessive IL-6 activation is linked to maladaptive remodeling and myocardial fibrosis [26,30]. IL-6 is a robust independent predictor of major adverse cardiovascular events (MACE) and mortality [31]. Patients in the highest quartiles of IL-6 exhibit a significantly higher risk of cardiovascular and all-cause mortality [27,31]. Serum levels of above 20 pg/mL within the first 24 h of STEMI are associated with higher frequencies of death, heart failure, and reinfarction [32].

Interleukin-1 beta is a potent pro-inflammatory cytokine and the “prototypical” alarmin of the innate immune system. Unlike IL-6, which is often used as a systemic marker of the “downstream” inflammatory cascade, interleukin-1 beta is an “upstream” mediator. Its production is tightly regulated by the NLRP3 inflammasome, which is activated by cellular debris (DAMPs) released from necrotic cardiomyocytes during an acute myocardial infarction (AMI) [33]. Interleukin-1 beta exists in a precursor form (pro-interleuktin-1 beta) that must be cleaved by the enzyme caspase-1 within the inflammasome to become biologically active. In AMI, this activation occurs within minutes to hours of ischemia-reperfusion. It is a primary driver of adverse ventricular remodeling (thinning of the infarct wall and dilation of the chamber), which is the precursor to heart failure. Persistent elevation of IL-1beta or its upstream regulators (caspase-1) is associated with a higher risk of major adverse cardiovascular events (MACE) and premature death, as demonstrated by a French multi-center study of1398 STEMI patients [31,34].

The landmark CANTOS trial demonstrated that specifically targeting interleukin-1 beta with the monoclonal antibody canakinumab significantly reduced recurrent cardiovascular events in patients with a prior AMI and persistent inflammation, confirming the cytokine as a causal driver of risk rather than just a bystander [14]. While interleukin-1 beta and IL-6 are both critical to the inflammatory cascade in AMI, they present very differently in a clinical lab setting. Understanding their “diagnostic windows” helps explain why one is a staple of research (and potential therapy) while the other is more commonly used as a measurable systemic marker. The smaller ASSAIL-MI trial (Acute Study of Sarilumab in Myocardial Infarction) and its predecessors, like the Map-IT study, represent a major shift in how we think about treating a heart attack. Instead of just opening the blocked artery, these trials asked: Can we stop the “second wave” of damage caused by the immune system? Patients who received the IL-6 inhibitor had a significantly higher myocardial salvage index (the amount of heart muscle saved relative to the area at risk) compared to the placebo group [35]. Tocilizumab successfully blunted the surge of high-sensitivity CRP and other inflammatory markers that typically spike 24 h post-MI. Microvascular obstruction was reduced in the tocilizumab group, but there was no significant difference in infarct size [35].

Moving into the “omics” and advanced biomarker era, TNF-alpha and IL-18 are two of the most scrutinized cytokines. While IL-1 beta and IL-6 form the primary signaling axis, TNF-alpha and IL-18 act as critical amplifiers of tissue damage and predictors of mechanical failure. TNF-alpha is one of the earliest cytokines to rise in AMI, often detectable within minutes of ischemia. It is unique because it is not just released by immune cells, but is also synthesized directly by ischemic cardiomyocytes [30]. High levels are associated with increased vulnerability to ventricular arrhythmias. It remodels ion channels, shortening the refractory period and creating a substrate for re-entry [36]. Interleukin-1 beta is a potent pro-inflammatory cytokine and the “prototypical” alarmin of the innate immune system. Unlike IL-6, which is often used as a systemic marker of the “downstream” inflammatory cascade, IL-1 beta is an “upstream” mediator. Its production is tightly regulated by the NLRP3 inflammasome, which is activated by cellular debris (DAMPs) released from necrotic cardiomyocytes during an acute myocardial infarction (AMI) [33].

IL-18 is a prohypertrophic inflammatory cytokine directly triggering cardiac inflammation and fibrosis. It stimulates the production of matrix metalloproteinases (MMPs), which “dissolve” the cardiac skeleton, leading to wall thinning and heart failure [37]. IL-18 is a critical downstream effector of inflammasome activation in AMI that remains elevated for months after the acute event, independently predicts adverse outcomes, including mortality and heart failure, and directly contributes to cardiac dysfunction and adverse remodeling as demonstrated by several small trials in AMI patients [25,38,39]. Unlike other cytokines, IL-18 is heavily expressed within the atherosclerotic plaque itself. Elevated plasma IL-18 post-AMI is specifically linked to the risk of recurrent plaque rupture and secondary infarctions [39]. Preclinical evidence strongly supports IL-18 as a therapeutic target, with IL-18BP and neutralizing antibodies demonstrating cardioprotection [40,41]. However, clinical trials of IL-18 blockade in AMI are still needed to translate these findings to patient care [39].

The most commonly used inflammatory markers in clinical practice for risk prediction after acute myocardial infarction are CRP/hsCRP, IL-6, and complete blood count (CBC)-derived markers, particularly the neutrophil-to-lymphocyte ratio (NLR). NLR is the most extensively studied CBC-derived marker. A meta-analysis of >16,000 ACS patients showed that high admission NLR was associated with 4.60-fold higher mortality in STEMI and 6.41-fold higher mortality in NSTEMI [42]. In another metanalysis of >28,756 patients, post-PCI NLR ≥ 3.88 was associated with larger infarct size on cardiac MRI and 2.60-fold higher risk of long-term MACE [43].

Other CBC-derived markers showing independent prognostic value include the systemic immune-inflammation index (SII), systemic inflammatory response index (SIRI), and pan-immune-inflammation value (PIV). In a smaller retrospective study of nearly 700 STEMI patients, elevated SII (HR 3.83), SIRI (HR 2.70), and PIV (HR 3.17) were independently associated with 1-year mortality after adjustment for clinical variables [44]. For reinfarction specifically, only the platelet-to-lymphocyte ratio (PLR) shows independent association (OR 1.59) [44].

Yet the recently released 2025 American College of Cardiology Scientific Statement recommends universal screening of hsCRP, stating that, while other inflammatory biomarkers also predict risk, their routine evaluation adds little to hsCRP predictive value [19]. Here a discussion is warranted regarding the usage of hsCRP or standard CRP with respect to sensitivity in different contexts, cost and availability factors. Standard CRP and hsCRP perform comparably for risk stratification after acute myocardial infarction, with excellent correlation (r = 0.99) and 98.6% diagnostic accuracy when both can detect CRP levels [45]. This is a critical distinction from primary prevention, where hsCRP’s superior sensitivity to detect low-grade inflammation especially at thresholds below 3 mg/L is essential.

In a prospective study of 344 patients with acute MI followed for a median of 6 years, Hofer et al. found that standard CRP and hsCRP had nearly identical prognostic performance in direct comparison between the two markers [45]. Both showed similar but modest AUC values for predicting mortality (standard CRP: 0.565 vs. hsCRP: 0.572) and major adverse cardiac events (standard CRP: 0.607 vs. hsCRP: 0.526) when measured during the acute phase. The correlation between the two assays was exceptional (r = 0.99, *p* < 0.001), and diagnostic accuracy was 98.6%. Longitudinal inflammatory risk assessment in the stable phase (not acute measurement) in the study revealed that standard CRP had 100% specificity and 100% negative predictive value for predicting long-term mortality, with Kaplan–Meier analysis showing significant survival benefit for patients at low residual inflammatory risk (*p* = 0.014). This suggests that standard CRP may be sufficient for post-MI risk stratification, particularly in the stable phase [45].

In acute MI, CRP levels typically rise substantially, often well above 10 mg/L, due to myocardial necrosis and the acute inflammatory response [46]. In a study of 1186 STEMI patients, those with high inflammatory response had hsCRP levels >33 mg/L (90th percentile), well within the detection range of standard CRP assays [47]. This contrasts sharply with primary prevention, where median CRP is approximately 2 mg/L and most individuals have levels <3 mg/L, requiring hsCRP’s enhanced sensitivity [48].

hsCRP remains the most extensively validated and clinically accessible inflammatory marker for both short- and long-term risk stratification after MI [19]. In the acute phase, elevated hsCRP predicts in-hospital mortality, while, in the stable phase (≥1 month post-MI), persistently elevated levels (≥2 mg/L) identify patients at higher risk for recurrent events [49]. However, when hsCRP is unavailable, standard CRP can identify patients at low residual inflammatory risk with high specificity, and serial measurements in the stable post-MI phase provide meaningful prognostic information [45]. Further, some authors recognize the potential of combining CRP with other inflammatory markers such as IL-6 to refine prognostic prediction [50]. Moreover, CRP is recognized as a potential tool for tailored risk stratification, enabling the identification of individuals with persistently elevated post-MI inflammatory markers who necessitate more intensive monitoring and therapeutic intervention, (i.e., with anti-inflammatory agents like colchicine or statins with dual lipid-lowering and anti-inflammatory properties) [46]. Within this framework, our subgroup-specific cut-offs are coherent. In diabetes, chronic low-grade inflammation and endothelial dysfunction likely lower the threshold at which an additional acute inflammatory surge translates into clinical events; smokers, with heightened systemic inflammation and pro-thrombotic milieu, plausibly behave similarly. In younger patients—who carry fewer competing comorbid risks—the signal-to-noise ratio of acute inflammation is higher, so relatively small CRP elevations have larger prognostic weight. In subacute presentations, longer pre-hospital ischemia and delayed reperfusion allow inflammatory pathways to mature; thus, even modest admission CRP may reflect a biologically advanced response. Together, these observations move beyond the “CRP is predictive” narrative and suggest a context-dependent interpretation: who the patient is and how they present determines what level of CRP matters.

### 4.2. CRP Is Most Informative Early—And Especially in Vulnerable Profiles

In our study, AUROC curves at 30, 90, and 180 days showed stable, moderate AUCs in the overall cohort, aligning with the prior literature that positions CRP as a useful but not standalone discriminator. Schiele et al. [51] have shown that adding CRP to clinical risk models can improve risk classification in acute coronary syndromes, while cautioning against one-size-fits-all thresholds. A meta-analysis of Liu et al. [11] focused on AMI cohorts treated with PCI confirm that CRP associates with in-hospital and short-term adverse outcomes, emphasizing the early hazard period where an acute-phase biomarker should be maximally informative. Our restriction of ROC analyses to short-term mortality fits both biology and evidence; CRP peaks within 48–72 h and tracks the intensity of the immediate sterile inflammatory response, whose excess is most tightly linked to early death and adverse remodeling [2].

The novel contribution is quantifying how much the threshold drops in specific clinical profiles. In diabetics, optimal cut-offs around ~5–6 mg/dL yielded the best discrimination across all time points, indicating that clinicians should not wait for CRP to enter the “high” range before taking notice. Smokers and subacute presentations showed optimal thresholds around ~9–10 mg/dL, and younger patients required less elevation than the overall population for meaningful risk separation. These subgroup effects are not merely statistical curiosities; they mirror mechanistic priors. Translational studies and reviews highlight that the same inflammatory modules that aid repair also drive extracellular-matrix turnover, infarct expansion, and ventricular dilation—processes amplified by metabolic inflammation and tobacco exposure [2,52]. In other words, equal CRP does not imply equal risk: context shapes how aggressively an inflammatory signal manifests clinically [53].

Clinically, this argues for a graded, profile-aware interpretation of admission CRP. A value of ~10 mg/dL may be reassuring in an older, multi-morbid patient if integrated with other signals, yet the same value in a 58-year-old smoker with subacute infarction should prompt heightened vigilance. Importantly, our AUCs are moderate by design; CRP should complement—not replace—established indices (hemodynamics, troponin/CK, LVEF, and ECG features). The potential value lies in early triage and triggering a lower threshold for intensified monitoring in the vulnerable profiles our study delineates.

### 4.3. How These Findings Align and Expand the Current Literature

Several strands of high-quality evidence support our interpretation. Mechanistic reviews outline the centrality of innate immune signaling after MI, including IL-1/IL-6 pathways that drive hepatocyte CRP synthesis; excessive or protracted activation predicts worse remodeling and heart failure [2,54]. Translational work links higher inflammatory activity to greater infarct size and adverse LV geometry, providing a substrate for CRP’s association with outcomes [55]. On the prognostic side, both Schiele et al. [51] and Lakhani et al. [56] consistently associate elevated CRP with mortality and heart failure, particularly early after the event.

Our hypothesis-generating findings extend this canon in two ways. First, we operationalize the concept that inflammation’s prognostic weight is time-dependent by focusing ROC analyses on short-term endpoints, where CRP’s biology is most relevant. This choice is consistent with pathophysiology and with empirical data showing the strongest associations in the first months post-MI [46,53]. Second, and more importantly, we demonstrate clinically meaningful, subgroup-specific cut-offs—a departure from prior studies that typically reported uniform thresholds or relied on tertiles/quartiles [12,57,58]. While our retrospective study is exploratory and requires validation in large prospective trials, our findings provide a practical framework for context-aware interpretation during emergency and early post-STEMI hospital care. We specifically show that diabetics, smokers, younger patients, and those with subacute presentations demonstrate risk at lower CRP levels.

These results also converse with therapeutic evidence. The CANTOS trial [14] proved that lowering inflammation via IL-1β inhibition reduces recurrent cardiovascular events in patients with prior MI and elevated inflammation parameters, independent of lipid lowering, thereby validating inflammation as a causal driver in atherosclerotic events. Subsequent analyses explored outcomes such as heart-failure hospitalization and provided further support for the IL-1β→IL-6→CRP axis as a modifiable pathway [59]. While our observational design was not able to test therapy, the subgroup sensitivity we report suggests who might benefit most from investigative anti-inflammatory strategies after STEMI: patients whose risk rises at relatively low CRP, such as those with diabetes or subacute presentation. In these groups, even modest inflammatory activation appears to have outsized clinical consequences—precisely the biology targeted by IL-1 pathway inhibition [60].

Finally, our data showing context-dependent cutoffs may have implications for risk-model calibration. Currently, the GRACE score is the gold-standard risk stratification tool for ACS patients, with robust validation across diverse populations. The original GRACE 2.0 achieves C-statistics of 0.77–0.86 for predicting in-hospital, 30-day, and 1-year mortality [61]. The 2025 ACC/AHA ACS guidelines recommend risk scores like GRACE for initial risk stratification, however, the guidelines highlight a limitation in the evidence regarding whether routine application of these scores translates into reduced cardiovascular events [62]. Furthermore, the incremental value of incorporating biomarkers into these risk models remains contentious, with conflicting data regarding improvements in event prediction. One study found that the addition of continuous high-sensitivity troponin in GRACE 3.0 further improved discrimination to 0.87–0.91 [61]. Another study found that adding high CRP (>22 mg/L) to the GRACE score modestly improved the C-statistic from 0.795 to 0.823 and allowed adequate reclassification in 12.2% of patients [51]. However, a study in AMI patients undergoing primary PCI found that biomarkers including high-sensitivity CRP did not provide additional benefit for mortality prediction beyond the GRACE score alone (AUC 0.870 vs. 0.868, *p* = 0.747) [63]. Schiele et al. [51] indicated that adding CRP can improve ACS risk prediction, but the field has lacked guidance on thresholds tailored to patient context. Our proposed, context-dependent CRP thresholds require external validation before clinical implementation, nonetheless, our results imply that clinicians may need to lower their vigilance threshold for high-risk monitoring when modest CRP elevations occur in specific patient populations, such as diabetics, smokers, younger patients, or subacute STEMI. Future studies should test whether incorporating profile-specific CRP thresholds into established scores (e.g., GRACE or TIMI) improves calibration and net reclassification, and whether dynamic measures (serial CRP or IL-6) further refine early risk determination.

## 5. Conclusions

This study is hypothesis-generating and suggests that admission CRP, beyond confirming its role as a predictor of adverse outcomes after STEMI, can identify vulnerable subgroups in whom risk emerges at much lower thresholds. In younger patients, diabetics, smokers, and those with subacute infarction, CRP values well below the conventional high-risk range already discriminate short-term mortality. This subgroup-specific sensitivity is a novel finding, emphasizing that the prognostic meaning of CRP is not uniform but context-dependent. Recognizing these lower cut-offs may help refine early risk stratification and guide closer surveillance in populations where modest inflammatory activation translates into disproportionate clinical risk.

## 6. Limitations

Several limitations merit consideration. First, the study was retrospective and conducted at a single, tertiary center, which may limit external validity and carries the risk of residual confounding. On the other hand, the non-randomized dataset represents the actual clinical practice in the state of Salzburg as a real-word study as opposed to randomized trials with a higher reproducibility but not necessarily better representation of the patient cohort constituting our STEMI population. Second, the ROC analyses were restricted to short-term outcomes, reflecting the acute-phase nature of CRP, and subgroup-specific cut-offs should therefore be regarded as hypothesis-generating rather than definitive. External validation in independent cohorts will be essential before clinical implementation. Third, cardiogenic shock and respiratory failure (intubation) were captured in one category of severe disease in our database but not separately. As no subanalysis of cardiogenic shock and/or respiratory failure was done, no CRP cutoffs for these specific entities were captured.

Several limitations related to CRP measurement must be noted. Only admission CRP values were analyzed as serial CRP measurements were not performed at standardized time points for all STEMI patients. This approach may have limited our ability to capture the dynamic inflammatory response that evolves over the course of acute myocardial infarction, as CRP typically peaks 48–72 h after symptom onset.

Finally, C-reactive protein was measured using standard assays rather than high-sensitivity C-reactive protein (hs-CRP), reflecting routine clinical practice in our ST-elevation myocardial infarction patients in our region. While standard CRP and hs-CRP demonstrate high correlation and similar diagnostic accuracy in acute myocardial infarction populations [64], the newly published American guidelines argue for the implementation of hs-CRP measurement in risk determination and stratification [19]. Due to availability and cost-effectiveness of standard CRP assays, our study offers insight into cardiovascular risk assessment, especially in special populations using widely available standard CRP assays. While our retrospective findings are exploratory, prospective studies are warranted to validate tailored CRP and other inflammation marker cutoffs, including hsCRP for risk assessment in specific STEMI sub-populations.

## Figures and Tables

**Table 1:** Baseline characteristics. Key: CRP = C-reactive protein; mg/dL = milligram per deciliter; BMI = body mass index; CHD = coronary heart disease; CPR = cardiopulmonary resuscitation; DES = drug eluting stent; CABG = coronary artery bypass grafting; AF = atrial fibrillation.

	Overall	CRP < 5 mg/dL	CRP 5–9.9 mg/dL	CRP 10–15 mg/dL	CRP > 15 mg/dL	*p*
**n (%)**						
Number	958 (100.0)	623 (100.0)	115 (100.0)	83 (100.0)	137 (100.0)	-
Gender (Male)	703 (73.4)	454 (72.9)	84 (73.0)	56 (67.5)	109 (79.6)	0.235
Age						
<50 years	104 (10.9)	70 (11.2)	19 (16.5)	7 (8.4)	8 (5.8)	0.047
50–69 years	546 (57.0)	368 (59.1)	63 (54.8)	38 (45.8)	77 (56.2)	0.132
>70 years	308 (32.2)	185 (29.7)	33 (28.7)	38 (45.8)	52 (38.0)	0.009
CVRF						
Arterial Hypertension	641 (66.9)	413 (66.3)	73 (63.5)	62 (74.7)	93 (67.9)	0.363
Dyslipidemia	666 (69.5)	451 (72.4)	80 (69.6)	60 (72.3)	75 (54.7)	0.001
Diabetes	156 (16.5)	87 (14.0)	24 (20.9)	12 (14.5)	33 (24.1)	0.014
Active Smoking	398 (41.5)	264 (42.4)	49 (42.6)	36 (43.4)	49 (35.8)	0.526
BMI ≥ 25 kg/m^2^	644 (67.2)	429 (68.9)	74 (64.3)	54 (65.1)	87 (63.5)	0.848
STEMI-Characteristics						
CHD—1 vessel	378 (39.5)	266 (42.7)	36 (31.3)	28 (33.7)	48 (35.0)	0.042
CHD—2 vessels	270 (28.2)	180 (28.9)	33 (28.7)	25 (30.1)	32 (23.4)	0.593
CHD—3 vessels	310 (32.4)	177 (28.4)	46 (40.0)	30 (36.1)	57 (41.6)	0.004
CPR	137 (14.3)	38 (6.1)	22 (19.1)	22 (26.5)	55 (40.1)	<0.001
Cardiogenic shock/						
Respiratory failure	163 (17.0)	39 (6.3)	26 (22.6)	32 (38.6)	66 (48.2)	<0.001
Fibrinolysis	24 (2.5)	7 (1.1)	6 (5.2)	5 (6.0)	6 (4.4)	0.002
DES implantation	877 (91.5)	596 (95.7)	101 (87.8)	71 (85.5)	109 (79.6)	<0.001
CABG indication	43 (4.5)	8 (1.3)	4 (3.5)	8 (9.6)	23 (16.8)	<0.001
Acute STEMI	554 (57.8)	373 (59.9)	57 (49.6)	44 (53.0)	80 (58.4)	0.158
Previous Disease						
MI	113 (11.8)	71 (11.4)	19 (16.5)	6 (7.2)	17 (12.4)	0.234
PCI or CABG	137 (14.3)	90 (14.4)	21 (18.3)	8 (9.6)	18 (13.1)	0.376
Heart Failure	55 (5.7)	30 (4.8)	10 (8.7)	5 (6.0)	10 (7.3)	0.319
Renal Insufficiency *	103 (10.8)	51 (8.2)	13 (11.3)	10 (12.0)	29 (21.2)	<0.001
AF	43 (4.5)	26 (4.2)	9 (7.8)	1 (1.2)	7 (5.1)	0.150
Cancer	91 (9.5)	51 (8.2)	15 (13.0)	9 (10.8)	16 (11.7)	0.277
**mean ± SD**						
Age (years)	63.4 ± 12.0	62.8 ± 11.9	62.7 ± 12.3	65.3 ± 12.6	65.9 ± 11.4	0.018
BMI (kg/m^2^)	27.2 ± 4.5	27.4 ± 4.6	27.1 ± 4.2	26.6 ± 4.2	27.3 ± 4.3	0.506
LVEF (%)	43.2 ± 9.8	45.6 ± 8.4	40.2 ± 11.1	38.6 ± 9.9	37.8 ± 10.9	<0.001
**median ± IQR**						
Laboratory Values						
Total cholesterol (mg/dL)	181.5 ± 63.0	187.0 ± 62.3	177.0 ± 69.0	183.0 ± 58.3	160.0 ± 76.0	<0.001
HDL (mg/dL)	49.0 ± 19.0	48.0 ± 18.3	49.5 ± 19.8	49.0 ± 16.8	49.0 ±21.3	0.217
Non-HDL (mg/dL)	132.0 ± 62.0	136.0 ± 61.5	129.0 ± 62.0	137.5 ± 50.3	112.0 ± 63.8	0.001
LDL (mg/dL)	107.0 ± 58.3	111.0 ± 56.3	98.0 ± 57.5	112.0 ± 52.0	86.5 ± 61.5	<0.001
Triglycerides/mg/dL)	111.0 ± 80.0	114.0 ± 84.0	108.0 ± 78.3	107.5 ± 70.0	118.0 ± 84.3	0.514
Troponin T max (ng/L)	3414.0 ± 5375.5	3031.0 ± 4684.0	5286.5 ± 6787.8	4599.0 ± 7211.0	4350.5 ± 7661.8	<0.001
Creatin kinase max (U/L)	1408.5 ± 2191.5	1194.0 ± 1768.8	1921.0 ± 2892.5	2089.5 ± 2755.8	2278.0 ± 3007.3	<0.001
HbA1c (%)	5.6 ± 0.6	5.5 ± 0.5	5.6 ± 0.9	5.7 ± 0.6	5.6 ± 0.8	0.284

* eGFR < 60 mL/min/1.73m^2^.

**Table 2:** ROC analysis of admission CRP for 30-day mortality. Key: Discriminatory performance of CRP for predicting 30-day mortality in the overall cohort and predefined subgroups (sex, age, diabetes, smoking, BMI, and STEMI presentation). Area under the curve (AUC), 95% confidence interval (CI), *p*-value, optimal cut-off (Youden index), sensitivity, and specificity are reported. * n = number of deaths.

	Value	Prediction	n *	AUC 95% CI	*p*-Value	Cut-off	Sensitivity	Specificity	Youden Index
A	CRP	30d mortality	57	0.628 0.539–0.720	0.001	11.55	0.47	0.82	0.29
B			38	0.623 0.510–0.737	0.010	11.40	0.47	0.81	0.28
C			19	0.648 0.492–0.804	0.032	12.35	0.47	0.88	0.35
D			27	0.601 0.457–0.744	0.076	9.65	0.56	0.81	0.37
E			30	0.645 0.527–0.763	0.009	14.50	0.47	0.86	0.33
F			13	0.678 0.522–0.833	0.034	5.35	0.85	0.62	0.46
G			42	0.633 0.526–0.739	0.004	14.45	0.43	0.88	0.31
H			18	0.692 0.545–0.840	0.006	9.65	0.61	0.80	0.41
I			39	0.598 0.485–0.712	0.041	14.50	0.41	0.86	0.27
J			34	0.658 0.536–0.780	0.002	9.95	0.56	0.80	0.36
K			16	0.635 0.470–0.800	0.070	14.50	0.50	0.87	0.37
L			27	0.655 0.537–0.772	0.007	6.10	0.63	0.68	0.31
M			30	0.600 0.463–0.737	0.065	11.55	0.50	0.83	0.33

**Table 3:** ROC analysis of admission CRP for 90-day mortality. Key: Discriminatory performance of CRP for predicting 90-day mortality in the overall cohort and predefined subgroups (sex, age, diabetes, smoking, BMI, and STEMI presentation). AUC, 95% CI, *p*-value, optimal cut-off (Youden index), sensitivity, and specificity are reported. * n = number of deaths.

	Value	Prediction	n *	AUC 95% CI	*p*-Value	Cut-off	Sensitivity	Specificity	Youden Index
A	CRP	90d mortality	66	0.653 0.568–0.738	<0.001	14.45	0.47	0.88	0.35
B			45	0.659 0.555–0.764	<0.001	14.45	0.49	0.86	0.35
C			21	0.646 0.497–0.794	0.027	12.35	0.48	0.88	0.36
D			33	0.677 0.540–0.794	0.001	9.65	0.64	0.82	0.46
E			33	0.628 0.512–0.745	0.016	14.50	0.46	0.86	0.32
F			15	0.714 0.571–0.857	0.007	5.35	0.87	0.62	0.49
G			48	0.644 0.544–0.745	0.001	14.45	0.46	0.89	0.35
H			20	0.680 0.533–0.826	0.007	9.65	0.60	0.80	0.40
I			46	0.640 0.536–0.744	0.002	14.35	0.48	0.87	0.35
J			40	0.687 0.577–0.798	<0.001	11.55	0.58	0.84	0.42
K			17	0.605 0.441–0.770	0.144	14.50	0.47	0.87	0.34
L			30	0.662 0.546–0.778	0.003	6.10	0.63	0.69	0.32
M			36	0.642 0.519–0.764	0.004	11.55	0.56	0.83	0.39

**Table 4:** ROC analysis of admission CRP for 180-day mortality. Key: Discriminatory performance of CRP for predicting 180-day mortality in the overall cohort and predefined subgroups (sex, age, diabetes, smoking, BMI, and STEMI presentation). AUC, 95% CI, *p*-value, optimal cut-off (Youden index), sensitivity, and specificity are reported. * n = number of deaths.

	Value	Prediction	n *	AUC 95% CI	*p*-Value	Cut-off	Sensitivity	Specificity	Youden Index
A	CRP	180d mortality	74	0.654 0.574–0.734	<0.001	14.35	0.46	0.88	0.34
B			52	0.655 0.559–0.751	<0.001	14.45	0.48	0.87	0.35
C			22	0.657 0.513–0.800	0.015	12.10	0.50	0.87	0.37
D			35	0.660 0.536–0.785	0.001	9.40	0.63	0.82	0.45
E			39	0.636 0.531–0.742	0.006	14.50	0.44	0.87	0.30
F			20	0.705 0.578–0.832	0.003	6.25	0.75	0.68	0.43
G			51	0.641 0.543–0.739	0.001	14.45	0.45	0.89	0.34
H			21	0.693 0.551–0.835	0.003	9.65	0.62	0.80	0.42
I			53	0.637 0.541–0.732	0.001	14.35	0.45	0.87	0.32
J			47	0.678 0.577–0.779	<0.001	11.55	0.55	0.84	0.39
K			18	0.625 0.465–0.785	0.075	14.50	0.50	0.87	0.37
L			32	0.640 0.526–0.755	0.008	6.10	0.59	0.69	0.28
M			42	0.661 0.551–0.771	0.001	11.50	0.57	0.84	0.41

## Data Availability

Data was captured by the corresponding author in a pseudoanonymized database and can be made available upon request.

## Abbreviations

The following abbreviations are used in this manuscript:

ANOVA	Analysis of Variance
AUC	Area Under the Curve
BMI	Body Mass Index
CABG	Coronary Artery Bypass Grafting
CHD	Coronary Heart Disease
CRP	C-Reactive Protein
DAMPs	Danger-associated molecular patterns
ECG	Electrocardiogram
HR	Hazard Ratio
ICH-GCP	International Council for Harmonization –Good Clinical Practice
IL-6	Interleukin-6
IQR	Interquartile Range
mg/dL	Milligram per deciliter
PCI	Percutaneous Coronary Intervention
ROC	Receiver operating characteristic
STEMI	ST-segment elevation myocardial infarction
YI	Youden Index
